# Bis(5-chloro­salicylato-κ*O*)bis­(1,10-phenanthroline-κ^2^ *N*,*N*′)cadmium(II)

**DOI:** 10.1107/S1600536808015687

**Published:** 2008-06-07

**Authors:** Decai Wen, Jing Xie, Xiurong Jiang

**Affiliations:** a Department of Chemistry, Longyan University, Longyan, Fujian 364000, People’s Republic of China

## Abstract

In the title complex, [Cd(C_7_H_4_ClO_3_)_2_(C_12_H_8_N_2_)_2_], the Cd atom is coordinated by two 5-chloro­salicylate ligands and two 1,10-phenanthroline ligands, displaying a distorted octa­hedral coordination geometry. The crystal structure is stabilized by O—H⋯O and C—H⋯O hydrogen bonds and π–π inter­actions between the 1,10-phenanthroline ligands and 5-chloro­salicylate ligands, with a centroid–centroid distance between neighbouring aromatic rings of 3.730 (1) Å.

## Related literature

For related literature, see: Lemoine *et al.* (2004 ▶); Melnik *et al.* (2001 ▶); Wen, Liu & Ribas (2007 ▶); Wen & Ying (2007 ▶); Wen, Ta *et al.* (2007 ▶); Yin *et al.* (2004 ▶); Zhu *et al.* (2003 ▶).
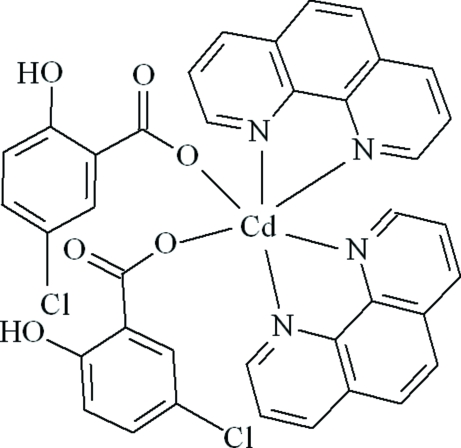


## Experimental

### 

#### Crystal data

[Cd(C_7_H_4_ClO_3_)_2_(C_12_H_8_N_2_)_2_]
*M* *_r_* = 815.91
Orthorhombic, 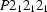

*a* = 10.812 (3) Å
*b* = 16.495 (4) Å
*c* = 18.862 (5) Å
*V* = 3363.9 (14) Å^3^
*Z* = 4
Mo *K*α radiation
μ = 0.86 mm^−1^
*T* = 293 (2) K
0.25 × 0.23 × 0.22 mm

#### Data collection

Rigaku R-AXIS RAPID IP diffractometer
Absorption correction: none
32955 measured reflections
7696 independent reflections
5812 reflections with *I* > 2σ(*I*)
*R*_int_ = 0.086

#### Refinement

*R*[*F* ^2^ > 2σ(*F* ^2^)] = 0.045
*wR*(*F* ^2^) = 0.086
*S* = 1.01
7696 reflections
461 parameters
H-atom parameters constrained
Δρ_max_ = 0.64 e Å^−3^
Δρ_min_ = −0.55 e Å^−3^
Absolute structure: Flack (1983 ▶), with 3402 Friedel pairs
Flack parameter: 0.00 (19)

### 

Data collection: *RAPID-AUTO* (Rigaku, 1998 ▶); cell refinement: *RAPID-AUTO*; data reduction: *RAPID-AUTO*; program(s) used to solve structure: *SHELXS97* (Sheldrick, 2008 ▶); program(s) used to refine structure: *SHELXL97* (Sheldrick, 2008 ▶); molecular graphics: *SHELXTL-Plus* (Sheldrick, 2008 ▶); software used to prepare material for publication: *SHELXL97*.

## Supplementary Material

Crystal structure: contains datablocks I, global. DOI: 10.1107/S1600536808015687/wn2261sup1.cif

Structure factors: contains datablocks I. DOI: 10.1107/S1600536808015687/wn2261Isup2.hkl

Additional supplementary materials:  crystallographic information; 3D view; checkCIF report

## Figures and Tables

**Table:** 

Cd1—O4	2.261 (3)
Cd1—O1	2.336 (4)
Cd1—N4	2.400 (3)
Cd1—N2	2.400 (4)
Cd1—N1	2.422 (3)
Cd1—N3	2.423 (3)

**Table:** 

O4—Cd1—O1	82.14 (12)
O4—Cd1—N4	125.46 (12)
O1—Cd1—N4	83.28 (13)
O4—Cd1—N2	86.00 (13)
O1—Cd1—N2	156.59 (12)
N4—Cd1—N2	87.33 (12)
O4—Cd1—N1	84.29 (12)
O1—Cd1—N1	129.12 (13)
N4—Cd1—N1	141.39 (12)
N2—Cd1—N1	69.12 (12)
O4—Cd1—N3	164.68 (11)
O1—Cd1—N3	97.76 (14)
N4—Cd1—N3	69.51 (11)
N2—Cd1—N3	98.95 (13)
N1—Cd1—N3	83.98 (12)

**Table 2:** Hydrogen-bond geometry (Å, °)

*D*—H⋯*A*	*D*—H	H⋯*A*	*D*⋯*A*	*D*—H⋯*A*
O3—H3⋯O2	0.82	1.76	2.504 (6)	149
O6—H6⋯O5	0.82	1.83	2.553 (6)	147
C15—H15*A*⋯O2	0.93	2.45	3.181 (6)	136
C36—H36*A*⋯O5	0.93	2.34	3.134 (6)	143
C24—H24*A*⋯O3^i^	0.93	2.42	3.185 (6)	140

Symmetry code: (i) 
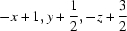
.

**Fig. 1.**
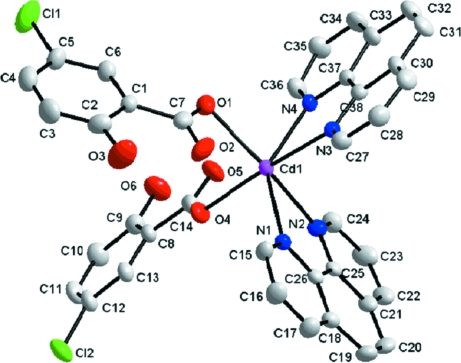
Molecular structure of the title complex with displacement ellipsoids drawn at the 30% probability level. H atoms have been omitted for clarity.

**Fig. 2.**
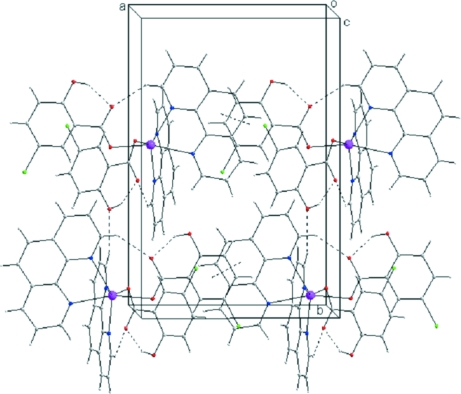
A packing view of the title complex, showing the O—H···O and C—H···O hydrogen bonds and the π–π interactions as dashed lines.

## supplementary crystallographic information

### Comment

Salicylic acid and its derivatives continue to attract attention because of their versatile coordination modes (Zhu *et al.*, 2003; Yin *et al.*, 2004; Wen, Liu & Ribas, 2007) and biological applications (Lemoine *et al.*, 2004). We report here the structure of a Cd (II) complex with the 5-chlorosalicylate ligand (Melnik *et al.*, 2001; Wen & Ying, 2007; Wen, Ta *et al.*, 2007). The title complex, Cd(C_7_H_4_ClO_3_)_2_ (C_12_H_8_N_2_)_2_, was synthesized under hydrothermal conditions.

The Cd atom is coordinated in a distorted octahedral coordination geometry by two O atoms from two 5-chlorosalicylate ligands and four N atoms from two 1,10-phenanthroline ligands (Fig. 1 and Table 1). The crystal structure is stabilized by O—H···O and C—H···O hydrogen bonds (Table 2), and π–π interactions between the 1,10-phenanthroline ligands and 5-chlorosalicylate ligands, with a centroid–centroid distance between neighbouring aromatic rings of 3.730 (1) Å (Fig. 2).

### Experimental

A mixture of Cd(NO_3_)_2_.6H_2_O (0.1 mmol), 1,10-phenanthroline (0.1 mmol), 5-chlorosalicylic acid (0.2 mmol) and distilled water (10 ml) was put into a Teflon-lined autoclave (20 ml) and then heated at 130 °C for 48 h. Colorless, block-like crystals of the title compound suitable for X-ray analysis were collected from the reaction mixture.

### Refinement

H atoms on C atoms were positioned geometrically and refined as riding, with C—H = 0.93 Å and *U*_iso_(H) = 1.2*U*_eq_(C). H atoms of hydroxyl groups were found in a difference Fourier map; they were then placed in calculated positions and refined as riding, with O—H = 0.82 Å and *U*_iso_(H) = 1.2*U*_eq_(O).

### Crystal data

**Table:**

[Cd(C_7_H_4_ClO_3_)_2_(C_12_H_8_N_2_)_2_]	*D*_x_ = 1.611 Mg m^−^^3^
*M**_r_* = 815.91	Mo *K*α radiation λ = 0.71073 Å
Orthorhombic, *P*2_1_2_1_2_1_	Cell parameters from 7696 reflections
*a* = 10.812 (3) Å	θ = 3.1–27.5º
*b* = 16.495 (4) Å	µ = 0.86 mm^−^^1^
*c* = 18.862 (5) Å	*T* = 293 (2) K
*V* = 3363.9 (14) Å^3^	Block, colourless
*Z* = 4	0.25 × 0.23 × 0.22 mm
*F*_000_ = 1640	

### Data collection

**Table:**

Rigaku R-AXIS RAPID IP diffractometer	5812 reflections with *I* > 2σ(*I*)
Radiation source: fine-focus sealed tube	*R*_int_ = 0.086
Monochromator: graphite	θ_max_ = 27.5º
*T* = 293(2) K	θ_min_ = 3.1º
ω scans	*h* = −12→14
Absorption correction: none	*k* = −21→21
32955 measured reflections	*l* = −24→24
7696 independent reflections	

### Refinement

**Table:**

Refinement on *F*^2^	Hydrogen site location: inferred from neighbouring sites
Least-squares matrix: full	H-atom parameters constrained
*R*[*F*^2^ > 2σ(*F*^2^)] = 0.045	*w* = 1/[σ^2^(*F*_o_^2^) + (0.0296*P*)^2^] where *P* = (*F*_o_^2^ + 2*F*_c_^2^)/3
*wR*(*F*^2^) = 0.086	(Δ/σ)_max_ = 0.001
*S* = 1.01	Δρ_max_ = 0.64 e Å^−^^3^
7696 reflections	Δρ_min_ = −0.55 e Å^−^^3^
461 parameters	Extinction correction: none
Primary atom site location: structure-invariant direct methods	Absolute structure: Flack (1983), with 3402 Friedel pairs
Secondary atom site location: difference Fourier map	Flack parameter: 0.00 (19)

### Special details

**Table:**

Geometry. All e.s.d.'s (except the e.s.d. in the dihedral angle between two l.s. planes) are estimated using the full covariance matrix. The cell e.s.d.'s are taken into account individually in the estimation of e.s.d.'s in distances, angles and torsion angles; correlations between e.s.d.'s in cell parameters are only used when they are defined by crystal symmetry. An approximate (isotropic) treatment of cell e.s.d.'s is used for estimating e.s.d.'s involving l.s. planes.
Refinement. Refinement of *F*^2^ against ALL reflections. The weighted *R*-factor *wR* and goodness of fit *S* are based on *F*^2^, conventional *R*-factors *R* are based on *F*, with *F* set to zero for negative *F*^2^. The threshold expression of *F*^2^ > σ(*F*^2^) is used only for calculating *R*-factors(gt) *etc*. and is not relevant to the choice of reflections for refinement. *R*-factors based on *F*^2^ are statistically about twice as large as those based on *F*, and *R*- factors based on ALL data will be even larger.

### Fractional atomic coordinates and isotropic or equivalent isotropic displacement parameters (Å^2^)

**Table:**

	*x*	*y*	*z*	*U*_iso_*/*U*_eq_	
Cd1	0.59780 (3)	0.543762 (17)	0.728736 (18)	0.03400 (8)	
N1	0.6215 (3)	0.42023 (19)	0.6606 (2)	0.0351 (8)	
N2	0.6379 (3)	0.57366 (19)	0.6064 (2)	0.0405 (9)	
N3	0.8074 (3)	0.51532 (19)	0.7668 (2)	0.0376 (8)	
N4	0.7042 (3)	0.66724 (19)	0.7571 (2)	0.0386 (9)	
O1	0.5104 (3)	0.5619 (2)	0.8409 (2)	0.0605 (10)	
O2	0.5242 (4)	0.4294 (3)	0.8289 (2)	0.0812 (13)	
O3	0.3784 (5)	0.3371 (2)	0.8932 (3)	0.1014 (17)	
H3	0.4380	0.3512	0.8693	0.122*	
O4	0.3969 (3)	0.5341 (2)	0.69660 (18)	0.0548 (8)	
O5	0.4036 (4)	0.6686 (2)	0.6992 (2)	0.0669 (10)	
O6	0.2125 (4)	0.7513 (2)	0.6663 (2)	0.0731 (12)	
H6	0.2842	0.7434	0.6786	0.088*	
C1	0.3742 (4)	0.4805 (2)	0.9087 (3)	0.0388 (11)	
C2	0.3237 (6)	0.4034 (3)	0.9218 (3)	0.0581 (15)	
C3	0.2220 (6)	0.3961 (4)	0.9663 (4)	0.0672 (17)	
H3A	0.1883	0.3451	0.9747	0.081*	
C4	0.1713 (5)	0.4616 (4)	0.9977 (3)	0.0624 (15)	
H4A	0.1029	0.4560	1.0272	0.075*	
C5	0.2221 (4)	0.5366 (3)	0.9854 (3)	0.0528 (13)	
C6	0.3213 (4)	0.5468 (3)	0.9411 (3)	0.0452 (11)	
H6A	0.3528	0.5984	0.9329	0.054*	
C7	0.4778 (5)	0.4913 (4)	0.8564 (3)	0.0526 (13)	
C8	0.2189 (4)	0.6063 (3)	0.6605 (3)	0.0405 (11)	
C9	0.1585 (5)	0.6802 (3)	0.6510 (3)	0.0493 (13)	
C10	0.0397 (5)	0.6807 (3)	0.6231 (3)	0.0572 (16)	
H10A	−0.0011	0.7297	0.6165	0.069*	
C11	−0.0181 (5)	0.6095 (3)	0.6050 (3)	0.0580 (15)	
H11A	−0.0976	0.6104	0.5863	0.070*	
C12	0.0422 (4)	0.5369 (3)	0.6149 (3)	0.0473 (12)	
C13	0.1593 (4)	0.5347 (3)	0.6422 (3)	0.0397 (11)	
H13A	0.1991	0.4852	0.6485	0.048*	
C14	0.3497 (4)	0.6014 (3)	0.6875 (3)	0.0484 (12)	
C15	0.6077 (5)	0.3456 (2)	0.6847 (3)	0.0438 (11)	
H15A	0.5841	0.3391	0.7318	0.053*	
C16	0.6260 (5)	0.2764 (3)	0.6442 (3)	0.0531 (14)	
H16A	0.6136	0.2252	0.6635	0.064*	
C17	0.6625 (5)	0.2852 (3)	0.5757 (3)	0.0556 (14)	
H17A	0.6760	0.2397	0.5476	0.067*	
C18	0.6800 (4)	0.3631 (3)	0.5471 (3)	0.0443 (12)	
C19	0.7225 (5)	0.3769 (3)	0.4766 (3)	0.0563 (14)	
H19A	0.7415	0.3327	0.4480	0.068*	
C20	0.7358 (5)	0.4521 (4)	0.4505 (3)	0.0573 (13)	
H20A	0.7661	0.4595	0.4048	0.069*	
C21	0.7036 (4)	0.5214 (3)	0.4930 (3)	0.0448 (12)	
C22	0.7083 (5)	0.6011 (3)	0.4667 (3)	0.0592 (15)	
H22A	0.7336	0.6109	0.4203	0.071*	
C23	0.6757 (6)	0.6634 (3)	0.5094 (3)	0.0614 (16)	
H23A	0.6759	0.7164	0.4924	0.074*	
C24	0.6420 (5)	0.6471 (3)	0.5792 (3)	0.0499 (14)	
H24A	0.6212	0.6906	0.6083	0.060*	
C25	0.6666 (4)	0.5101 (3)	0.5636 (3)	0.0353 (10)	
C26	0.6559 (4)	0.4298 (2)	0.5917 (3)	0.0342 (10)	
C27	0.8556 (4)	0.4427 (3)	0.7750 (3)	0.0476 (11)	
H27A	0.8071	0.3979	0.7637	0.057*	
C28	0.9760 (5)	0.4291 (3)	0.7999 (3)	0.0541 (14)	
H28A	1.0064	0.3767	0.8052	0.065*	
C29	1.0470 (5)	0.4942 (3)	0.8160 (3)	0.0561 (14)	
H29A	1.1280	0.4868	0.8312	0.067*	
C30	0.9987 (4)	0.5725 (3)	0.8097 (3)	0.0442 (12)	
C31	1.0676 (4)	0.6433 (3)	0.8284 (3)	0.0568 (15)	
H31A	1.1500	0.6379	0.8416	0.068*	
C32	1.0166 (5)	0.7175 (3)	0.8272 (3)	0.0576 (15)	
H32A	1.0624	0.7623	0.8416	0.069*	
C33	0.8916 (5)	0.7276 (2)	0.8039 (3)	0.0449 (11)	
C34	0.8324 (5)	0.8039 (3)	0.8018 (3)	0.0583 (15)	
H34A	0.8747	0.8501	0.8164	0.070*	
C35	0.7141 (4)	0.8102 (3)	0.7785 (4)	0.0566 (14)	
H35A	0.6740	0.8601	0.7774	0.068*	
C36	0.6538 (4)	0.7391 (3)	0.7561 (3)	0.0498 (14)	
H36A	0.5730	0.7435	0.7396	0.060*	
C37	0.8229 (4)	0.6599 (2)	0.7804 (3)	0.0358 (10)	
C38	0.8774 (4)	0.5804 (2)	0.7848 (2)	0.0363 (10)	
Cl1	0.15573 (18)	0.61989 (11)	1.02771 (12)	0.1006 (7)	
Cl2	−0.02954 (12)	0.44667 (9)	0.58967 (9)	0.0661 (4)	

### Atomic displacement parameters (Å^2^)

**Table:**

	*U*^11^	*U*^22^	*U*^33^	*U*^12^	*U*^13^	*U*^23^
Cd1	0.02979 (13)	0.03431 (13)	0.03791 (16)	−0.00051 (14)	−0.00110 (16)	−0.00186 (15)
N1	0.034 (2)	0.0349 (16)	0.037 (2)	−0.0032 (15)	0.0010 (17)	−0.0025 (15)
N2	0.049 (2)	0.0334 (17)	0.039 (2)	−0.0031 (16)	−0.0037 (18)	0.0030 (16)
N3	0.0365 (17)	0.0416 (17)	0.035 (2)	−0.0004 (15)	0.000 (2)	−0.0025 (18)
N4	0.0321 (17)	0.0408 (17)	0.043 (3)	−0.0007 (15)	0.0025 (17)	−0.0043 (17)
O1	0.053 (2)	0.088 (3)	0.041 (2)	−0.014 (2)	0.0066 (17)	0.001 (2)
O2	0.079 (3)	0.103 (3)	0.061 (3)	0.018 (2)	0.021 (2)	−0.019 (2)
O3	0.140 (4)	0.051 (2)	0.113 (4)	−0.002 (3)	0.036 (4)	−0.015 (2)
O4	0.0339 (15)	0.066 (2)	0.065 (2)	0.008 (2)	−0.0101 (18)	−0.0056 (18)
O5	0.0520 (19)	0.072 (2)	0.077 (3)	−0.022 (2)	−0.012 (2)	−0.0070 (19)
O6	0.076 (3)	0.0439 (19)	0.100 (4)	−0.0039 (19)	−0.018 (3)	−0.004 (2)
C1	0.038 (3)	0.044 (2)	0.034 (3)	0.0016 (19)	0.000 (2)	0.0017 (19)
C2	0.075 (4)	0.048 (3)	0.051 (4)	−0.001 (3)	−0.002 (3)	−0.001 (3)
C3	0.070 (4)	0.060 (3)	0.071 (5)	−0.017 (3)	0.004 (4)	0.019 (3)
C4	0.051 (3)	0.077 (4)	0.060 (4)	−0.001 (3)	0.007 (3)	0.018 (4)
C5	0.047 (3)	0.056 (3)	0.055 (3)	0.013 (3)	0.010 (2)	0.001 (3)
C6	0.046 (2)	0.043 (2)	0.047 (3)	0.000 (3)	0.003 (2)	0.011 (3)
C7	0.047 (3)	0.072 (3)	0.039 (3)	0.001 (3)	−0.005 (2)	−0.006 (3)
C8	0.030 (2)	0.048 (3)	0.043 (3)	−0.003 (2)	−0.001 (2)	−0.001 (2)
C9	0.049 (3)	0.047 (3)	0.052 (4)	0.000 (2)	0.001 (3)	0.001 (2)
C10	0.051 (3)	0.049 (3)	0.072 (5)	0.011 (3)	−0.005 (3)	0.010 (3)
C11	0.034 (3)	0.069 (3)	0.071 (4)	0.003 (3)	−0.012 (3)	0.005 (3)
C12	0.031 (2)	0.054 (3)	0.056 (3)	−0.007 (2)	−0.003 (2)	0.011 (3)
C13	0.031 (2)	0.041 (2)	0.048 (3)	0.000 (2)	−0.004 (2)	0.009 (2)
C14	0.035 (2)	0.066 (3)	0.043 (3)	−0.008 (3)	0.000 (2)	0.001 (3)
C15	0.050 (3)	0.036 (2)	0.046 (3)	−0.001 (2)	0.008 (3)	0.0002 (19)
C16	0.065 (4)	0.033 (2)	0.062 (4)	0.000 (2)	0.008 (3)	0.001 (2)
C17	0.064 (4)	0.046 (3)	0.057 (4)	0.003 (3)	0.008 (3)	−0.007 (3)
C18	0.042 (3)	0.047 (3)	0.044 (3)	−0.003 (2)	−0.005 (2)	−0.009 (2)
C19	0.063 (3)	0.062 (3)	0.044 (4)	−0.005 (3)	0.001 (3)	−0.019 (3)
C20	0.064 (3)	0.079 (3)	0.029 (3)	−0.006 (3)	0.000 (2)	−0.007 (3)
C21	0.048 (3)	0.057 (3)	0.029 (3)	−0.011 (2)	−0.006 (2)	0.005 (2)
C22	0.069 (4)	0.073 (4)	0.035 (3)	−0.019 (3)	−0.008 (3)	0.009 (3)
C23	0.083 (4)	0.048 (3)	0.053 (4)	−0.011 (3)	−0.014 (3)	0.015 (3)
C24	0.055 (3)	0.037 (2)	0.057 (4)	−0.001 (2)	−0.005 (3)	0.004 (2)
C25	0.029 (2)	0.042 (2)	0.035 (3)	−0.0043 (19)	−0.008 (2)	−0.003 (2)
C26	0.029 (2)	0.039 (2)	0.034 (3)	−0.0043 (18)	−0.007 (2)	0.0000 (19)
C27	0.051 (2)	0.044 (3)	0.048 (3)	0.005 (2)	−0.004 (3)	−0.004 (3)
C28	0.059 (3)	0.048 (3)	0.055 (4)	0.013 (2)	−0.004 (3)	0.003 (2)
C29	0.043 (3)	0.070 (3)	0.055 (4)	0.009 (3)	−0.012 (3)	0.004 (3)
C30	0.034 (2)	0.049 (3)	0.049 (3)	−0.002 (2)	−0.006 (2)	0.005 (2)
C31	0.037 (3)	0.064 (3)	0.070 (4)	−0.002 (2)	−0.021 (3)	−0.001 (3)
C32	0.045 (3)	0.058 (3)	0.070 (4)	−0.010 (3)	−0.017 (3)	0.000 (3)
C33	0.040 (2)	0.048 (2)	0.046 (3)	−0.010 (2)	−0.003 (3)	0.002 (2)
C34	0.054 (3)	0.044 (3)	0.077 (4)	−0.012 (2)	−0.004 (3)	−0.014 (3)
C35	0.053 (3)	0.039 (2)	0.078 (4)	0.007 (2)	−0.008 (3)	−0.009 (3)
C36	0.039 (2)	0.045 (2)	0.066 (4)	0.004 (2)	−0.007 (2)	−0.001 (2)
C37	0.034 (2)	0.041 (2)	0.033 (3)	−0.0050 (18)	0.003 (2)	−0.002 (2)
C38	0.036 (2)	0.044 (2)	0.029 (3)	−0.0012 (18)	−0.0002 (19)	0.0018 (18)
Cl1	0.1042 (14)	0.0923 (12)	0.1054 (17)	0.0301 (10)	0.0478 (12)	0.0013 (11)
Cl2	0.0482 (7)	0.0668 (8)	0.0834 (11)	−0.0173 (7)	−0.0204 (7)	0.0048 (8)

### Geometric parameters (Å, °)

**Table:**

Cd1—O4	2.261 (3)	C12—Cl2	1.745 (5)
Cd1—O1	2.336 (4)	C13—H13A	0.9300
Cd1—N4	2.400 (3)	C15—C16	1.388 (6)
Cd1—N2	2.400 (4)	C15—H15A	0.9300
Cd1—N1	2.422 (3)	C16—C17	1.360 (8)
Cd1—N3	2.423 (3)	C16—H16A	0.9300
N1—C15	1.320 (5)	C17—C18	1.406 (7)
N1—C26	1.361 (6)	C17—H17A	0.9300
N2—C24	1.316 (5)	C18—C26	1.410 (6)
N2—C25	1.359 (5)	C18—C19	1.425 (7)
N3—C27	1.316 (5)	C19—C20	1.343 (8)
N3—C38	1.357 (5)	C19—H19A	0.9300
N4—C36	1.304 (5)	C20—C21	1.438 (7)
N4—C37	1.362 (5)	C20—H20A	0.9300
O1—C7	1.252 (6)	C21—C25	1.403 (7)
O2—C7	1.250 (6)	C21—C22	1.407 (7)
O3—C2	1.356 (6)	C22—C23	1.352 (8)
O3—H3	0.8200	C22—H22A	0.9300
O4—C14	1.233 (6)	C23—C24	1.393 (8)
O5—C14	1.272 (5)	C23—H23A	0.9300
O6—C9	1.342 (6)	C24—H24A	0.9300
O6—H6	0.8200	C25—C26	1.433 (6)
C1—C6	1.377 (6)	C27—C28	1.402 (7)
C1—C2	1.405 (7)	C27—H27A	0.9300
C1—C7	1.504 (7)	C28—C29	1.355 (7)
C2—C3	1.389 (8)	C28—H28A	0.9300
C3—C4	1.348 (8)	C29—C30	1.397 (6)
C3—H3A	0.9300	C29—H29A	0.9300
C4—C5	1.374 (8)	C30—C38	1.399 (6)
C4—H4A	0.9300	C30—C31	1.430 (6)
C5—C6	1.370 (7)	C31—C32	1.343 (7)
C5—Cl1	1.743 (5)	C31—H31A	0.9300
C6—H6A	0.9300	C32—C33	1.430 (7)
C8—C13	1.390 (6)	C32—H32A	0.9300
C8—C9	1.394 (6)	C33—C37	1.412 (6)
C8—C14	1.506 (6)	C33—C34	1.414 (6)
C9—C10	1.388 (7)	C34—C35	1.356 (7)
C10—C11	1.372 (7)	C34—H34A	0.9300
C10—H10A	0.9300	C35—C36	1.407 (6)
C11—C12	1.376 (7)	C35—H35A	0.9300
C11—H11A	0.9300	C36—H36A	0.9300
C12—C13	1.368 (6)	C37—C38	1.441 (6)
			
O4—Cd1—O1	82.14 (12)	N1—C15—C16	124.1 (5)
O4—Cd1—N4	125.46 (12)	N1—C15—H15A	117.9
O1—Cd1—N4	83.28 (13)	C16—C15—H15A	117.9
O4—Cd1—N2	86.00 (13)	C17—C16—C15	118.5 (4)
O1—Cd1—N2	156.59 (12)	C17—C16—H16A	120.7
N4—Cd1—N2	87.33 (12)	C15—C16—H16A	120.7
O4—Cd1—N1	84.29 (12)	C16—C17—C18	120.0 (5)
O1—Cd1—N1	129.12 (13)	C16—C17—H17A	120.0
N4—Cd1—N1	141.39 (12)	C18—C17—H17A	120.0
N2—Cd1—N1	69.12 (12)	C17—C18—C26	117.3 (5)
O4—Cd1—N3	164.68 (11)	C17—C18—C19	123.1 (5)
O1—Cd1—N3	97.76 (14)	C26—C18—C19	119.5 (4)
N4—Cd1—N3	69.51 (11)	C20—C19—C18	121.6 (5)
N2—Cd1—N3	98.95 (13)	C20—C19—H19A	119.2
N1—Cd1—N3	83.98 (12)	C18—C19—H19A	119.2
C15—N1—C26	117.9 (4)	C19—C20—C21	120.3 (5)
C15—N1—Cd1	126.1 (3)	C19—C20—H20A	119.9
C26—N1—Cd1	116.0 (3)	C21—C20—H20A	119.9
C24—N2—C25	118.1 (4)	C25—C21—C22	118.0 (5)
C24—N2—Cd1	124.7 (3)	C25—C21—C20	119.5 (4)
C25—N2—Cd1	117.0 (3)	C22—C21—C20	122.5 (5)
C27—N3—C38	118.0 (4)	C23—C22—C21	119.4 (5)
C27—N3—Cd1	125.6 (3)	C23—C22—H22A	120.3
C38—N3—Cd1	116.3 (2)	C21—C22—H22A	120.3
C36—N4—C37	118.6 (4)	C22—C23—C24	119.0 (5)
C36—N4—Cd1	124.6 (3)	C22—C23—H23A	120.5
C37—N4—Cd1	116.7 (2)	C24—C23—H23A	120.5
C7—O1—Cd1	101.9 (3)	N2—C24—C23	123.7 (5)
C2—O3—H3	109.5	N2—C24—H24A	118.2
C14—O4—Cd1	111.8 (3)	C23—C24—H24A	118.2
C9—O6—H6	109.5	N2—C25—C21	121.8 (4)
C6—C1—C2	118.7 (4)	N2—C25—C26	118.4 (4)
C6—C1—C7	120.4 (4)	C21—C25—C26	119.8 (4)
C2—C1—C7	120.8 (4)	N1—C26—C18	122.1 (4)
O3—C2—C3	121.1 (5)	N1—C26—C25	118.9 (4)
O3—C2—C1	119.4 (5)	C18—C26—C25	119.1 (5)
C3—C2—C1	119.4 (5)	N3—C27—C28	123.6 (4)
C4—C3—C2	121.3 (5)	N3—C27—H27A	118.2
C4—C3—H3A	119.4	C28—C27—H27A	118.2
C2—C3—H3A	119.4	C29—C28—C27	118.3 (4)
C3—C4—C5	119.0 (5)	C29—C28—H28A	120.9
C3—C4—H4A	120.5	C27—C28—H28A	120.9
C5—C4—H4A	120.5	C28—C29—C30	120.2 (4)
C6—C5—C4	121.8 (5)	C28—C29—H29A	119.9
C6—C5—Cl1	120.3 (4)	C30—C29—H29A	119.9
C4—C5—Cl1	117.9 (4)	C29—C30—C38	117.7 (4)
C5—C6—C1	119.8 (5)	C29—C30—C31	122.7 (4)
C5—C6—H6A	120.1	C38—C30—C31	119.6 (4)
C1—C6—H6A	120.1	C32—C31—C30	121.8 (4)
O2—C7—O1	123.4 (5)	C32—C31—H31A	119.1
O2—C7—C1	118.3 (5)	C30—C31—H31A	119.1
O1—C7—C1	118.3 (5)	C31—C32—C33	119.9 (5)
C13—C8—C9	119.6 (4)	C31—C32—H32A	120.1
C13—C8—C14	118.2 (4)	C33—C32—H32A	120.1
C9—C8—C14	122.1 (4)	C37—C33—C34	117.2 (4)
O6—C9—C10	118.6 (5)	C37—C33—C32	120.2 (4)
O6—C9—C8	122.2 (5)	C34—C33—C32	122.7 (4)
C10—C9—C8	119.2 (5)	C35—C34—C33	120.3 (4)
C11—C10—C9	120.7 (5)	C35—C34—H34A	119.8
C11—C10—H10A	119.6	C33—C34—H34A	119.8
C9—C10—H10A	119.6	C34—C35—C36	118.1 (4)
C10—C11—C12	119.7 (5)	C34—C35—H35A	121.0
C10—C11—H11A	120.2	C36—C35—H35A	121.0
C12—C11—H11A	120.2	N4—C36—C35	124.0 (4)
C13—C12—C11	120.9 (5)	N4—C36—H36A	118.0
C13—C12—Cl2	119.4 (4)	C35—C36—H36A	118.0
C11—C12—Cl2	119.7 (4)	N4—C37—C33	121.8 (4)
C12—C13—C8	120.0 (5)	N4—C37—C38	119.0 (4)
C12—C13—H13A	120.0	C33—C37—C38	119.1 (4)
C8—C13—H13A	120.0	N3—C38—C30	122.2 (4)
O4—C14—O5	124.8 (5)	N3—C38—C37	118.5 (4)
O4—C14—C8	119.0 (4)	C30—C38—C37	119.2 (4)
O5—C14—C8	116.2 (5)		
			
O4—Cd1—N1—C15	−88.3 (4)	C14—C8—C13—C12	−177.4 (5)
O1—Cd1—N1—C15	−13.3 (4)	Cd1—O4—C14—O5	−4.5 (7)
N4—Cd1—N1—C15	127.7 (4)	Cd1—O4—C14—C8	175.4 (4)
N2—Cd1—N1—C15	−176.2 (4)	C13—C8—C14—O4	−4.4 (8)
N3—Cd1—N1—C15	81.8 (4)	C9—C8—C14—O4	178.1 (5)
O4—Cd1—N1—C26	93.7 (3)	C13—C8—C14—O5	175.5 (5)
O1—Cd1—N1—C26	168.7 (3)	C9—C8—C14—O5	−2.0 (8)
N4—Cd1—N1—C26	−50.3 (4)	C26—N1—C15—C16	0.0 (7)
N2—Cd1—N1—C26	5.8 (3)	Cd1—N1—C15—C16	−177.9 (4)
N3—Cd1—N1—C26	−96.1 (3)	N1—C15—C16—C17	1.1 (8)
O4—Cd1—N2—C24	92.3 (4)	C15—C16—C17—C18	−0.5 (8)
O1—Cd1—N2—C24	32.7 (6)	C16—C17—C18—C26	−1.2 (8)
N4—Cd1—N2—C24	−33.6 (4)	C16—C17—C18—C19	177.6 (5)
N1—Cd1—N2—C24	177.7 (4)	C17—C18—C19—C20	178.6 (5)
N3—Cd1—N2—C24	−102.3 (4)	C26—C18—C19—C20	−2.6 (8)
O4—Cd1—N2—C25	−92.4 (3)	C18—C19—C20—C21	−1.9 (8)
O1—Cd1—N2—C25	−152.0 (3)	C19—C20—C21—C25	4.6 (7)
N4—Cd1—N2—C25	141.8 (3)	C19—C20—C21—C22	−175.9 (5)
N1—Cd1—N2—C25	−7.0 (3)	C25—C21—C22—C23	−1.0 (7)
N3—Cd1—N2—C25	73.0 (3)	C20—C21—C22—C23	179.5 (5)
O4—Cd1—N3—C27	7.9 (8)	C21—C22—C23—C24	2.0 (8)
O1—Cd1—N3—C27	96.5 (4)	C25—N2—C24—C23	−0.9 (7)
N4—Cd1—N3—C27	176.3 (5)	Cd1—N2—C24—C23	174.3 (4)
N2—Cd1—N3—C27	−99.9 (4)	C22—C23—C24—N2	−1.1 (8)
N1—Cd1—N3—C27	−32.2 (4)	C24—N2—C25—C21	2.0 (6)
O4—Cd1—N3—C38	−168.0 (4)	Cd1—N2—C25—C21	−173.6 (3)
O1—Cd1—N3—C38	−79.4 (3)	C24—N2—C25—C26	−176.7 (4)
N4—Cd1—N3—C38	0.4 (3)	Cd1—N2—C25—C26	7.7 (5)
N2—Cd1—N3—C38	84.1 (3)	C22—C21—C25—N2	−1.1 (7)
N1—Cd1—N3—C38	151.8 (3)	C20—C21—C25—N2	178.5 (4)
O4—Cd1—N4—C36	0.0 (5)	C22—C21—C25—C26	177.6 (4)
O1—Cd1—N4—C36	−75.4 (4)	C20—C21—C25—C26	−2.8 (7)
N2—Cd1—N4—C36	83.1 (4)	C15—N1—C26—C18	−1.9 (6)
N1—Cd1—N4—C36	134.1 (4)	Cd1—N1—C26—C18	176.3 (3)
N3—Cd1—N4—C36	−176.3 (4)	C15—N1—C26—C25	177.5 (4)
O4—Cd1—N4—C37	175.3 (3)	Cd1—N1—C26—C25	−4.4 (5)
O1—Cd1—N4—C37	99.9 (3)	C17—C18—C26—N1	2.4 (7)
N2—Cd1—N4—C37	−101.6 (3)	C19—C18—C26—N1	−176.4 (4)
N1—Cd1—N4—C37	−50.6 (4)	C17—C18—C26—C25	−176.9 (4)
N3—Cd1—N4—C37	−1.0 (3)	C19—C18—C26—C25	4.3 (6)
O4—Cd1—O1—C7	75.3 (3)	N2—C25—C26—N1	−2.1 (6)
N4—Cd1—O1—C7	−157.4 (4)	C21—C25—C26—N1	179.1 (4)
N2—Cd1—O1—C7	135.6 (4)	N2—C25—C26—C18	177.2 (4)
N1—Cd1—O1—C7	−0.7 (4)	C21—C25—C26—C18	−1.5 (6)
N3—Cd1—O1—C7	−89.2 (3)	C38—N3—C27—C28	−1.5 (8)
O1—Cd1—O4—C14	83.4 (4)	Cd1—N3—C27—C28	−177.3 (4)
N4—Cd1—O4—C14	7.4 (4)	N3—C27—C28—C29	−0.4 (9)
N2—Cd1—O4—C14	−76.4 (4)	C27—C28—C29—C30	1.9 (9)
N1—Cd1—O4—C14	−145.8 (4)	C28—C29—C30—C38	−1.6 (9)
N3—Cd1—O4—C14	174.0 (5)	C28—C29—C30—C31	177.7 (6)
C6—C1—C2—O3	−176.7 (5)	C29—C30—C31—C32	−174.7 (6)
C7—C1—C2—O3	7.7 (8)	C38—C30—C31—C32	4.6 (9)
C6—C1—C2—C3	0.4 (8)	C30—C31—C32—C33	−2.8 (9)
C7—C1—C2—C3	−175.2 (5)	C31—C32—C33—C37	−1.7 (8)
O3—C2—C3—C4	176.6 (6)	C31—C32—C33—C34	179.7 (6)
C1—C2—C3—C4	−0.5 (10)	C37—C33—C34—C35	0.6 (8)
C2—C3—C4—C5	−0.4 (9)	C32—C33—C34—C35	179.3 (6)
C3—C4—C5—C6	1.4 (9)	C33—C34—C35—C36	−0.7 (9)
C3—C4—C5—Cl1	−179.2 (5)	C37—N4—C36—C35	−0.7 (8)
C4—C5—C6—C1	−1.4 (8)	Cd1—N4—C36—C35	174.5 (4)
Cl1—C5—C6—C1	179.1 (4)	C34—C35—C36—N4	0.8 (9)
C2—C1—C6—C5	0.5 (7)	C36—N4—C37—C33	0.7 (7)
C7—C1—C6—C5	176.1 (5)	Cd1—N4—C37—C33	−174.9 (4)
Cd1—O1—C7—O2	22.3 (7)	C36—N4—C37—C38	177.1 (5)
Cd1—O1—C7—C1	−155.3 (4)	Cd1—N4—C37—C38	1.5 (6)
C6—C1—C7—O2	179.1 (5)	C34—C33—C37—N4	−0.6 (7)
C2—C1—C7—O2	−5.4 (8)	C32—C33—C37—N4	−179.3 (5)
C6—C1—C7—O1	−3.2 (7)	C34—C33—C37—C38	−177.0 (5)
C2—C1—C7—O1	172.4 (5)	C32—C33—C37—C38	4.3 (7)
C13—C8—C9—O6	−178.5 (5)	C27—N3—C38—C30	1.8 (7)
C14—C8—C9—O6	−1.0 (8)	Cd1—N3—C38—C30	178.0 (4)
C13—C8—C9—C10	−0.2 (8)	C27—N3—C38—C37	−176.0 (4)
C14—C8—C9—C10	177.3 (5)	Cd1—N3—C38—C37	0.2 (5)
O6—C9—C10—C11	178.4 (6)	C29—C30—C38—N3	−0.2 (8)
C8—C9—C10—C11	0.1 (9)	C31—C30—C38—N3	−179.6 (5)
C9—C10—C11—C12	0.1 (10)	C29—C30—C38—C37	177.5 (5)
C10—C11—C12—C13	−0.2 (9)	C31—C30—C38—C37	−1.8 (7)
C10—C11—C12—Cl2	−178.2 (5)	N4—C37—C38—N3	−1.2 (7)
C11—C12—C13—C8	0.0 (8)	C33—C37—C38—N3	175.4 (4)
Cl2—C12—C13—C8	178.1 (4)	N4—C37—C38—C30	−179.0 (5)
C9—C8—C13—C12	0.2 (7)	C33—C37—C38—C30	−2.5 (7)

### Hydrogen-bond geometry (Å, °)

**Table:**

*D*—H···*A*	*D*—H	H···*A*	*D*···*A*	*D*—H···*A*
O3—H3···O2	0.82	1.76	2.504 (6)	149
O6—H6···O5	0.82	1.83	2.553 (6)	147
C15—H15A···O2	0.93	2.45	3.181 (6)	136
C36—H36A···O5	0.93	2.34	3.134 (6)	143
C24—H24A···O3^i^	0.93	2.42	3.185 (6)	140

Symmetry codes: (i) −*x*+1, *y*+1/2, −*z*+3/2.
